# Plasma HDL Reduces Nonesterified Fatty Acid Hydroperoxides Originating from Oxidized LDL: a Mechanism for Its Antioxidant Ability

**DOI:** 10.1007/s11745-013-3779-1

**Published:** 2013-03-14

**Authors:** Mari Kotosai, Sachiko Shimada, Mai Kanda, Namiko Matsuda, Keiko Sekido, Yoshibumi Shimizu, Akira Tokumura, Toshiyuki Nakamura, Kaeko Murota, Yoshichika Kawai, Junji Terao

**Affiliations:** 1 Department of Food Science, Institute of Health Biosciences, University of Tokushima Graduate School, Kuramoto-cho 3-18-15, Tokushima, 770-8503 Japan; 2 Department of Nursing Education, Institute of Health Biosciences, University of Tokushima Graduate School, Kuramoto-cho 3-18-15, Tokushima, 770-8503 Japan; 3 Department of Pharmaceutical Health Chemistry, University of Tokushima Graduate School, Kuramoto-cho 3-18-15, Tokushima, 770-8503 Japan; 4 Present Address: Keiko Kekido, Graduate School of Health Sciences, Kobe University, Kobe, 654-0142 Japan; 5 Present Address: Kaeko Murota, Department of Life Sciences, School of Science and Engineering, Kinki University, Osaka, 577-8502 Japan; 6 Present Address: Laboratory of Food and Biodynamics, Graduate School of Bioagricultural Sciences, Nagoya University, Nagoya, 464-8601 Japan

**Keywords:** HDL, Nonesterified fatty acid hydroperoxides, Atherosclerosis, Oxidized LDL, Apolipoprotein A-1, Platelet activating factor-acetyl hydrolase

## Abstract

The antioxidant property of plasma high-density lipoprotein (HDL) is thought to be involved in potential anti-atherogenic effects but the exact mechanism is not known. We aimed to reveal the contribution of HDL on the elimination of lipid hydroperoxides (LOOH) derived from oxidized low-density lipoprotein (LDL). Oxidized LDL prepared by copper ion-induced oxidation contained nonesterified fatty acid hydroperoxides (FFA-OOH) and lysophosphatidylcholine (lysoPtdCho), in addition to cholesteryl ester hydroperoxides (CE-OOH) and phosphatidylcholine hydroperoxides (PtdCho-OOH). A platelet-activating factor-acetylhydrolase (PAF-AH) inhibitor suppressed formation of FFA-OOH and lysoPtdCho in oxidized LDL. Among LOOH species, FFA-OOH was preferentially reduced by incubating oxidized LDL with HDL. HDL exhibited selective FFA-OOH reducing ability if it was mixed with a liposomal solution containing FFA-OOH, CE-OOH and PtdCho-OOH. Two-electron reduction of the hydroperoxy group to the hydroxy group was confirmed by the formation of 13-hydroxyoctadecadienoic acid from 13-hydroperoxyoctadecadienoic acid in HPLC analyses. This reducing effect was also found in apolipoprotein A-1 (apoA-1). FFA-OOH released from PtdCho-OOH due to PAF-AH activity in oxidized LDL undergo two-electron reduction by the reducing ability of apoA1 in HDL. This preferential reduction of FFA-OOH may participate in the mechanism of the antioxidant property of HDL.

## Introduction

Plasma high-density lipoprotein (HDL) is a complex of proteins and lipids. In this complex, apoprotein A-1 (apoA-1) is the major protein constituent, and free cholesterol, esterified cholesterol and phospholipids are the major lipid classes [1]. HDL has been believed to act as an anti-atherosclerotic factor. The mechanism of action is thought to involve the promotion of the efflux of cholesterol from macrophage-derived foam cells in the artery wall, thereby inhibiting the progression of atherosclerosis [2]. This prompts the first step of the reverse transport of cholesterol by HDL from extrahepatic tissues to the liver.

An alternative way for HDL to exert its anti-atherogenic function is its antioxidant property. Formation of oxidized low-density lipoprotein (LDL) has long been recognized as the initial event of atherosclerosis [3, 4]. That is, reactive oxygen species (ROS) generated via enzymatic or non-enzymatic means attack the unsaturated lipids of LDL to produce “seeding molecules” (i.e., lipid hydroperoxides (LOOH) including cholesteryl ester hydroperoxides (CE-OOH), and phosphatidylcholine hydroperoxides (PtdCho-OOH)) as primary oxidation products [5, 6]. LOOH are stable per se, but they are readily cleaved to short-chain carbonyls such as 4-hydroxynonenal, and short-chain carbonyls containing phospholipids (phospholipid core aldehydes), in the presence of heme or non-heme iron [7, 8]. These reactive carbonyls seem to be responsible for the modification of apo-B proteins in LDL, resulting in oxidized LDL followed by uptake by macrophages through scavenger receptors [9, 10].

The antioxidant property of HDL was reported first by Bowry et al. [11]. They claimed that HDL attenuated the buildup of oxidized LDL by acting as a major carrier of LOOH in blood plasma. In recent years, HDL has been assumed to prevent the formation of oxidized LDL by eliminating seeding molecules from LDL [12]. For example, Tselepis et al. [13] showed that platelet activating factor-acetylhydrolase (PAF-AH) in HDL could remove phospholipid core aldehydes because of its phospholipase A_2_ activity. In contrast, paraoxonase-1 (PON-1) has been proposed to be a critical factor for the hydrolysis and reduction of the peroxidized phospholipids present in LDL [14, 15]. Nevertheless, Marathe et al. [16] and Kriska et al. [17] clarified that PAF-AH but not PON-1 contributed to the activity of phospholipase A_2_ for PtdCho-OOH in the antioxidant property of HDL. Garner et al. [18] examined the effect of HDL on LOOH accumulated in oxidized LDL. They concluded that apo-A1 in HDL can convert CE-OOH to their hydroxy derivatives by the reducing ability of methionine residues in apoA-1. Thereafter, Navab et al. [19] suggested that apoA-1 inhibits the formation of oxidized LDL by removing seeding molecules, including nonesterified fatty acid hydroperoxides (FFA-OOH). These studies suggested that the major HDL apolipoprotein has antioxidant activity independent of PON-1 or PAF-AH.

Increasing numbers of epidemiological studies have supported the idea that HDL is an essential preventive factor in the progress of atherosclerosis [20]. However, the exact mechanism of action of its antioxidant property is incompletely understood. In the present study, we aimed to clarify the target molecule for elimination and/or hydrolysis by HDL among the LOOH species present in LDL, and to clarify the constituent responsible for the antioxidant ability of HDL. We applied quantitative thin-layer chromatography (TLC) analyses for evaluating the changes of LOOH species by the reaction of oxidized LDL with HDL. We found FFA-OOH release from PtdCho-OOH to be the most plausible compound for the reducing activity of HDL in its antioxidant effect.

## Materials and Methods

Human studies on the preparation of LDL and HDL were approved by the Ethical Committee of the University of Tokushima (Tokushima, Japan).

### Materials

1-Palmitoyl-2-linoleoyl-*sn*-glycero-3-phosphocholine (PL-PtdCho), linoleic acid (9*Z*,12*Z*-octadecadienoic acid), and cholesteryl linoleate (cholesteryl 9*Z*,12*Z*-octadecadienoate) were purchased from Sigma-Aldrich (St. Louis, MO, USA). PtdCho-OOH were prepared by the photosensitized oxidation of PL-PtdCho according to the method of Terao et al. [21] with slight modification. PtdCho-OOH were isolated by preparative TLC analyses using RP-18 F254 plates (thickness, 1 mm; Merck, Whitehouse Station, NJ, USA) with a developing solvent of chloroform/methanol/water (3:8:2 by vol). Linoleic acid hydroperoxides (LNA-OOH) were also prepared by the photosensitized oxidation of linoleic acid according to the same method as that for PtdCho-OOH. A developing solvent of hexane/diethyl ether (7:3, by vol) was applied for preparative TLC (silica gel 60 F254s; thickness, 2 mm; Merck). CE-OOH were prepared by 2,2′-azobis(2,4-dimethylvaleronitrile)-induced radical chain oxidation of cholesteryl linoleate [22]. CE-OOH were isolated by preparative TLC analyses using silica gel 60F254 s plates (thickness, 2 mm; Merck) with a developing solvent of hexane/diethyl ether (7:3, by vol). All compounds were checked for purity by analytical TLC [23]. The hydroperoxide contents of LNA-OOH and CE-OOH were determined using an iodometric assay [24]. PtdCho-OOH content was measured by the method of Bartlett [25]. 13-Hydroperoxyoctadecadienoic acid (13-HPODE) was obtained from Cayman Chemicals (Ann Arbor, MI, USA) and 13-hydroxyoctadecadienoic acid (13-HODE) was prepared by the reduction of 13-HPODE with sodium borohydride [26]. Pefabloc (4-[2-aminoethyl]benzenesulfonyl fluoride) was obtained from Pierce (Rockford, IL, USA). Chloramine-T, a buffered aqueous solution of apoA-1 from human plasma, dimyristoyl-*sn*-glycero-3-phosphocholine (DM-PtdCho) and *N*,*N*,*N*′,*N*′-tetramethyl-*p*-phenylenediamine dihydrochloride (TMPD) were obtained from Sigma-Aldrich. All other reagents were of analytical grade without purification.

### Preparation of LDL and HDL

Peripheral venous blood was drawn from healthy subjects after fasting overnight. Serum was obtained immediately by centrifugation at 3,500 rpm for 20 min at 4 °C. To obtain lipoprotein fractions, serum was subjected to further density gradient centrifugation [27]. Serum was adjusted to relative density (*d*) of 1.21 with KBr and overlaid with saline (*d* = 1.008), and centrifugation undertaken (80,000 rpm for 40 min at 4 °C). After collecting the LDL fraction (yellow layer), further centrifugation was carried out for 6 h and the HDL fraction separated from the lipoprotein-eliminated plasma fraction. The purity of each fraction was confirmed by sodium dodecyl sulfate–polyacrylamide gel electrophoresis (SDS-PAGE; data not shown). Each lipoprotein fraction was dialyzed at 4 °C for 24 h against Chelex 100-treated phosphate-buffered saline (PBS) [28]. Protein concentration in the solution was determined using the Bradford method [29]. The solution was stored under nitrogen gas at 4 °C for ≤10 days.

### Measurement of PAF-AH Activity

The PAF-AH activities of serum, LDL and HDL were measured using a PAF-AH Assay kit (Cayman Chemicals) using 2-thio PAF as the substrate [30].

### Copper Ion-Induced Oxidation of LDL and Preparation of Lipid Extracts

LDL solution was diluted by Chelex 100-treated PBS buffer (0.8 mg protein/mL) and incubated at 37 °C after the addition of cupric sulfate (final concentration, 5 μΜ). At each time interval, an aliquot was removed and lipids extracted using the method of Bligh and Dyer [31]. In the experiment focusing on PAF-AH inhibition, the PAF-AH inhibitor pefabloc (final concentration, 1.0 mM) was added to the LDL solution, which was then diluted by Chelex 100-treated PBS buffer (1.0 mg protein/mL) and incubated at 37 °C for 4 h. The LDL solution was dialyzed against Chelex 100-treated PBS at 4 °C for 12 h. The solution was then subjected to the procedure for copper ion-induced oxidation of LDL as described above.

### Preparation of Oxidized LDL

LDL solution was diluted by Chelex 100-treated PBS (0.8 mg protein/mL) and oxidation initiated by the addition of cupric sulfate (final concentration, 5 μΜ). After incubation at 37 °C for 4 h, the solution was dialyzed at 4 °C for 12 h against Chelex 100-treated PBS. The solution obtained was used immediately as oxidized LDL.

### Incubation of Oxidized LDL with HDL

Oxidized LDL solution was mixed with HDL solution to adjust the total volume to 1.0 mL (final LDL concentration: 0.3 mg protein/mL; final HDL concentration: 1.0, 2.0, and 4.0 mg protein/mL). The mixed solution was incubated at 37 °C for 6 h. After the incubation, the total lipids of each sample were extracted with chloroform containing 1 mM 2,6-di-*tert*-butyl-*p*-cresol/methanol (2:1, by vol) according to the method of Bligh and Dyer [31]. The extract was resolved in the solvent of chloroform/methanol (2:1, by vol) and subjected to quantitative TLC analyses.

### Incubation of Liposomal Suspension Containing LOOH with HDL

Chloroform solutions of DM-PtdCho and cholesterol were mixed in a glass test tube. Solutions of CE-OOH, PtdCho-OOH and LNA-OOH were also added and the solvent removed with a stream of nitrogen followed by evaporation under vacuum. The residue was dispersed in 0.4 mM of Tris–HCl buffer (0.1 M, pH 7.4, containing 0.135 M KCl and 5 mM CaCl_2_). The final concentration of each lipid was as follows in mM: DM-PtdCho, 5; cholesterol, 2.5; CE-OOH, 0.25; PtdCho-OOH, 0.25; LNA-OOH, 0.25. The suspension was vortex-mixed for 1 min followed by ultrasonic irradiation in an Astrason Ultrasonifier (Misonix, Farmingdale, NY, USA) for 30 s. The resulting multi-lamellar liposomal suspension was added to HDL solution to adjust the final volume to 1.33 mL with Tris–HCl buffer (final HDL concentration of 1.0, 2.0, 4.0 mg protein/mL). After incubation at 37 °C for 6 h, the total lipids of the solution were extracted with chloroform containing 1 mM 2,6-di-*tert*-butyl-*p*-cresol/methanol (2:1 v/v) according to the method of Bligh and Dyer [31]. To compare the effect of HDL on LNA-OOH with that of apoA-1, liposomes containing LNA-OOH with DM-PtdCho and cholesterol were prepared using the same procedure as that described above (in mM: LNA-OOH, 0.5; DM-PtdCho, 10; cholesterol, 5). To 100 μL of the liposomal solution, 600 μL of apoA-1 solution or HDL solution was added to adjust the final concentration to 0.5 mg protein/mL. For treatment with chloramine-T, a buffer solution of apoA-1 or HDL (1.0 ml/mL) was mixed with chloramine-T (0.5 mM) and incubated at 37 °C for 1 h. Then the chloramine-T-treated solution was added to the 100 μL liposomal suspension to adjust the final volume to 600 μL (final concentration of apoA-1 and HDL: 0.5 mg/mL). After incubation at 37 °C for 6 h, total lipids were extracted as described above. The extract was resolved in the solvent of chloroform/methanol (2:1, by vol) and subjected to quantitative TLC analyses.

### Quantitative TLC Analyses for LOOH and Lysophosphatidylcholine (lysoPtdCho) in Oxidized LDL and a Liposomal Suspension

Total lipids extracted from the reaction mixture were concentrated and applied to TLC plates using Linomat 5 (Camag, Muttenz, Switzerland). Reversed-phase TLC analyses were done with reversed-phase TLC plates (silica gel RP-18 F254; thickness, 0.25 mm; Merck Darmstadt, Berlin, Germany) and a developing solvent of chloroform/methanol/water (20:70:4, by vol). Normal-phase TLC analyses were undertaken with absorptive TLC plates (silica gel 60F254; thickness, 0.25 mm; Merck Darmstadt) and a solvent of hexane/diethyl ether/acetic acid (70:30:1, by vol). Detection of LOOH was achieved by exposure of the TLC plate to *N*,*N*,*N*′,*N*′-tetramethyl-p-phenylenediamine hydrochloride (TMPD) reagent [32]. LysoPtdCho was detected by the use of primuline reagent [33] with a developing solvent of chloroform/methanol/hexane/acetone/acetic acid/water (40:20:20:5:1.3:2, by vol). These reagents were sprayed uniformly onto the plate using a TLC/high-performance TLC sprayer (Camag). Each band on the plate was monitored using Image Capture 2D (Liponics, Tokyo, Japan) and quantified by use of the software for TLC (Just TLC: Liponics).

### HPLC Analyses of NEFA-OOH and their Hydroxyl Derivatives

HPLC was applied for the analysis of oxidized FFA involving hydroperoxides and their hydroxy derivatives. Lipid extracts were obtained from the reaction mixture of 13-HPODE or LNA-OOH (1 mM) containing a liposomal suspension (DM-PtdCho, 20 mM; cholesterol, 10 mM) with HDL (1.0 mg/mL) after incubation at 37 °C for 6 h. Then it was applied to a reversed-phase HPLC system (Liquid Chromatograph LC-10AS and UV–VIS detector SPD-10A; Shimadzu, Kyoto, Japan) equipped with a column of TSK gel ODS-80Ts (250 × 4.6 mm i.d.; Tosoh, Tokyo, Japan) and an eluting solvent of 0.1 % acetic acid/acetonitrile/tetrahydrofuran (52:30:18, by vol) [34]. The eluent was monitored by UV absorption at 235 nm with a flow rate of 1.0 mL/min.

### HPLC Analyses of ApoA-1 and Its Oxidized Derivatives in HDL

HDL (0.3 mg protein/mL) was treated with 30 μM chloramine-T at 37 °C for 1 h. In a separate experiment, HDL (3 mg protein/mL) was treated with a liposomal suspension containing 25 mM DM-PtdCho and 12.5 mM cholesterol as well as 1.25 mM LNA-OOH at 37 °C for 24 h. After the reaction, each sample was applied to a reversed-phase HPLC system (Liquid Chromatograph LC-20AD and SPD-20A; Shimadzu) with a column of TSK gel ODS-80Ts and a gradient solvent system of solvent A (water containing 0.1 % trifluoroacetic acid (TFA) and solvent B (acetonitrile containing 0.1 % TFA); 25 % (0–10 min), 25–45 % (10–15 min), 45–55 % (15–47 min), 55–95 % (47–57 min), 95–100 % (57–58 min) [35]. The eluent was monitored by UV absorption at 214 nm at a flow rate of 0.5 mL/min.

## Results

### Participation of PAF-AH in the Profile of LOOH in Oxidized LDL

**Fig. 1:**
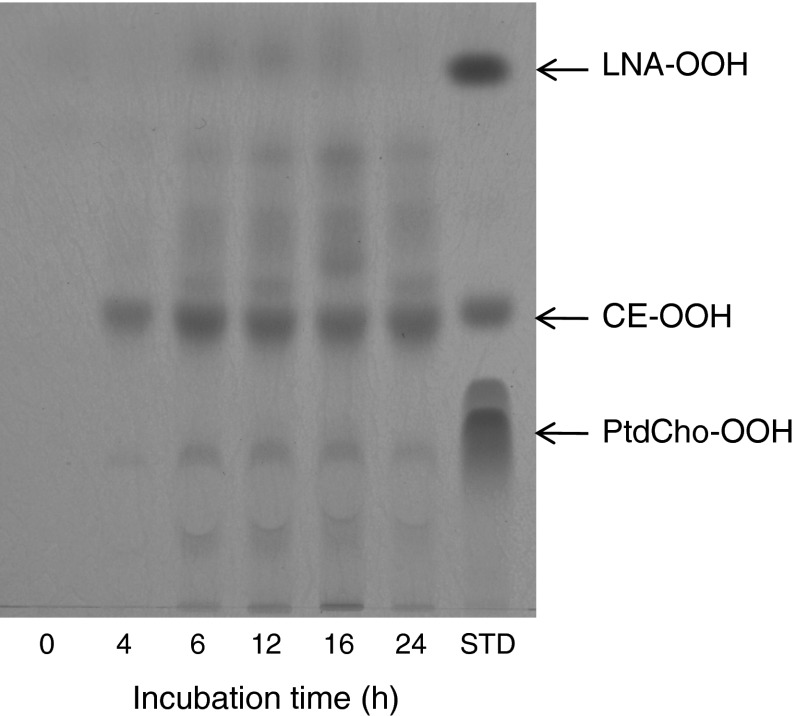
Detection of LOOH species in oxidized LDL by reversed-phase TLC analyses. LDL solution was diluted by Chelex 100-treated PBS buffer (0.8 mg protein/mL) and incubated at 37 °C after the addition of cupric sulfate (final concentration, 5 μM). At each time interval, lipids were extracted from the aliquot and analyzed using reversed-phase TLC plates (RP-18F254; Merck) with an eluting solvent of chloroform/methanol/water (20:70:4, by vol).* Bands* were detected by spraying with TMPD reagent

**Fig. 2:**
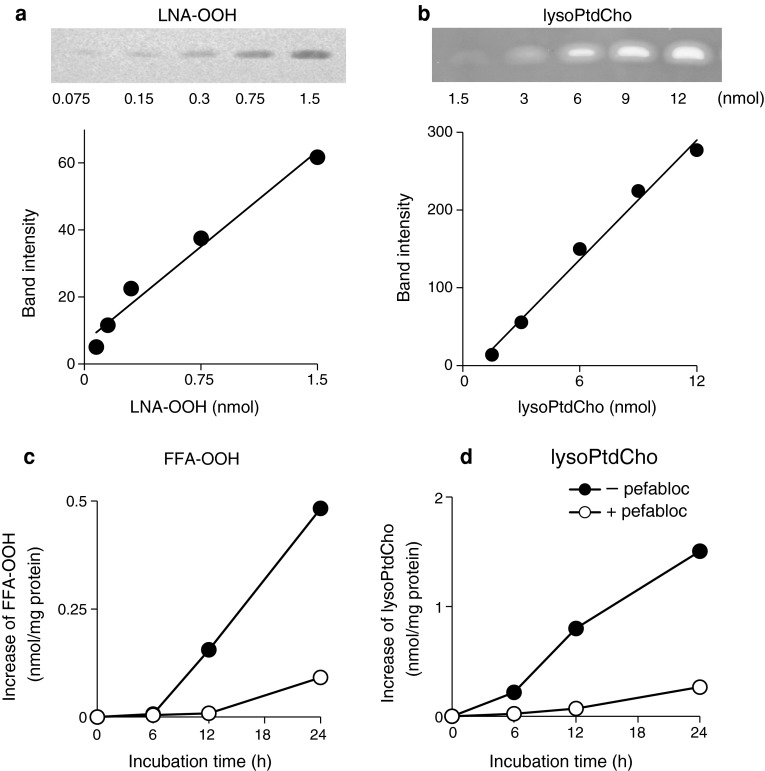
Effect of a PAF-AH inhibitor on the formation of NEFA-OOH and lysoPtdCho in the copper ion-induced oxidation of LDL. **a** *Standard curve* of LNA-OOH for determination of the contents of FFA-OOH, **b** *standard curve *of L-α-lysoPtdCho from egg yolk (Sigma–Aldrich) for determination of the content of lysoPtdCho. **c**, **d** Time course of the formation of NEFA-OOH and lysoPtdCho during the oxidation of LDL in the presence and absence of the PAF-AH inhibitor pefabloc. **a** LNA-OOH was determined by using normal-phase TLC plate silica gel (60F254; 0.25-mm thick; Merck) with a developing solvent of hexane/diethyl ether/acetic acid (70:30:1, by vol).* Bands* were detected by spraying with TMPD reagent. **b** LysoPtdCho was determined using the same normal TLC plate and using a developing solvent of chloroform/methanol/hexane/acetone/acetic acid/water (40:20:20:5:1.3:2, by vol).* Bands* were detected with primuline reagent. C, D: The inhibitor pefabloc (final concentration, 1.0 mM) was added to the LDL solution (1.0 mg protein/mL), and incubated at 37 °C for 4 h. The LDL solution was dialyzed against Chelex 100-treated PBS at 4 °C for 12 h. Then the solution was subjected to copper ion-induced oxidation of LDL under the same condition shown in Fig. 1. FFA-OOH was quantified using the* standard curve* of LNA-OOH shown in **a**. LysoPtdCho was quantified by the* standard curve* of lysoPtdCho shown in **b**

We used TMPD reagent for detection of the hydroperoxy group on reversed-phase TLC analyses of the lipid extracts obtained from oxidized LDL. The band corresponding to CE-OOH occupied most of the TMPD-positive bands of oxidized LDL at all time intervals (Fig. 1), indicating that CE-OOH was the major LOOH species of oxidized LDL. In contrast, FFA-OOH and PtdCho-OOH were minor components of the oxidized LDL because the bands corresponding to LNA-OOH and PtdCho-OOH were pale compared with that of CE-OOH. This TLC method was used for the quantitative analyses of FFA-OOH and lysoPtdCho because these two lipid species are the hydrolysis products of PtdCho-OOH during the incubation of oxidized LDL. Quantitative analysis was performed with a normal-phase TLC plate instead of a reversed-phase TLC plate because of the appearance of sharp bands in normal-phase one. We prepared standard curves of LNA-OOH (for FFA-OOH) and lysoPtdCho using normal-phase TLC plates, a spraying reagent of TMPD (LNA-OOH) and primuline (lysoPtdCho), and software for TLC analyses (Fig. 2a). The time courses of accumulation of FFA-OOH and lysoPtdCho with and without the addition of a PAF-AH inhibitor were obtained using these standard curves (Fig. 2b). Without the inhibitor, levels of both hydrolysis products increased with increasing incubation time. Interestingly, inhibitor treatment clearly suppressed the accumulation of FFA-OOH and lysoPtdCho. This result suggested that these two hydrolysis products were generated by the action of PAH-AH for PtdCho-OOH formed in oxidized LDL. In fact, the PAF-AH activities of serum, HDL and LDL were 0.17, 0.38 and 16.6 nmol/min/mg protein, respectively. It was therefore confirmed that the high PAF-AH activity of LDL hydrolyzes PtdCho-OOH through its phospholipase A_2_ activity, resulting in the accumulation of FFA-OOH and lysoPtdCho in oxidized LDL.

### Specificity of LOOH Species in Oxidized LDL for the Reducing Activity of HDL

**Fig. 3:**
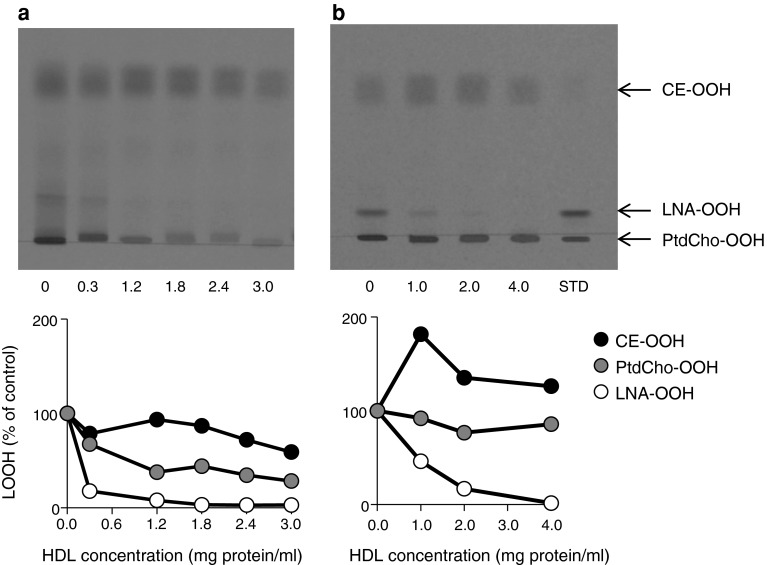
Effect of HDL on the contents of LOOH accumulated in oxidized LDL and incorporated into a liposomal suspension. **a** Oxidized LDL, **b** liposomal suspension. An oxidized LDL solution was mixed with HDL solution to adjust the total volume at 1.0 mL. After incubation of the mixture at 37 °C for 6 h, total lipids of each sample were extracted and subjected to quantitative TLC analyses. In the case of the liposomal suspension, chloroform solutions of DM-PtdCho and cholesterol were mixed with the solutions of CE-OOH and PtdCho-OOH as well as LNA-OOH, and the solvent removed with a stream of nitrogen followed by evaporation under vacuum. A multi-lamellar liposomal suspension containing DM-PtdCho (5 mM), cholesterol (2.5 mM), CE-OOH (0.25 mM), PtdCho-OOH (0.25 mM) and LNA-OOH (0.25 mM) was prepared by ultrasonication and then added to the HDL solution. After incubation at 37 °C for 6 h, the total lipids of the solution were extracted and subjected to quantitative TLC analyses using normal-phase TLC and a solvent system of hexane/diethyl ether/acetic acid (70:30:1, by vol).* Bands* were detected using the TMPD reagent

**Fig. 4:**
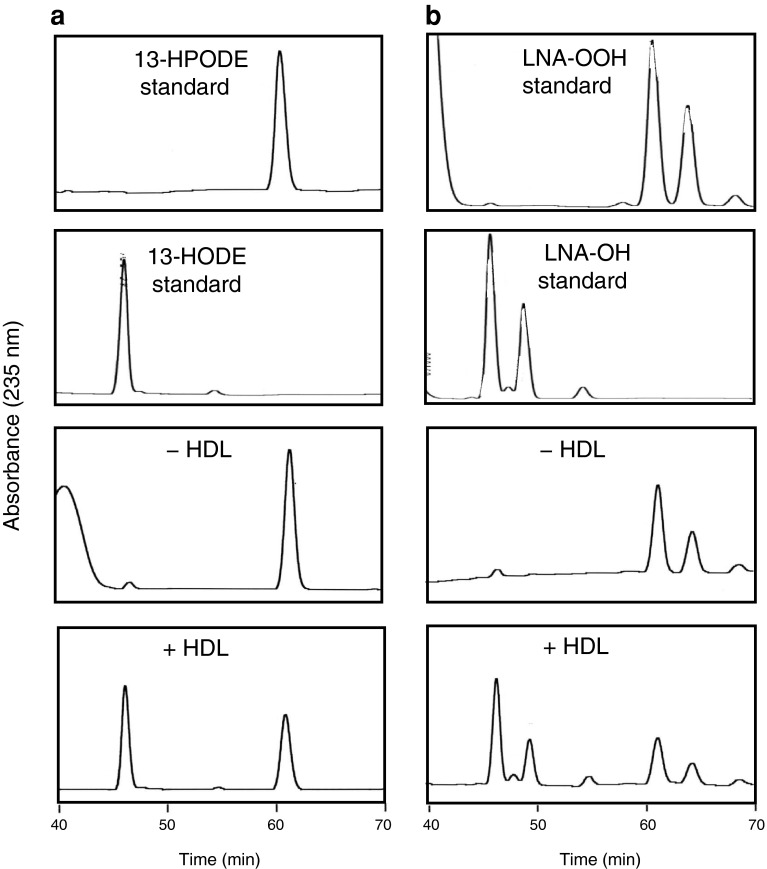
HDL-dependent reduction of HPODE isomers in a liposomal suspension. **a** HPLC analyses of 13-HPODE after incubation with or without HDL. **b** HPLC analyses of LNA-OOH after incubation with or without HDL. Liposomes containing 13-HPODE were prepared using the same procedure described in Fig. 3 (13-HPODE/LNA-OOH; 1 mM, DM-PtdCho; 20 mM, cholesterol; 10 mM). HDL solution was added to adjust the final concentration to 1.0 mg protein/mL. After incubation at 37 °C for 6 h, total lipids were extracted as described above and subjected to HPLC analyses

Different amounts of HDL were mixed with oxidized LDL and the total lipids extracted after incubation for 6 h. Normal-phase TLC plates sprayed with TMPD reagent showed one major band corresponding to CE-OOH and two minor bands corresponding to FFA-OOH and PtdCho-OOH in the absence of HDL (Fig. 3a). The bands for FFA-OOH and PtdCho-OOH were diminished by incubation with HDL depending on its content; the band for CE-OOH was only slightly changed by the incubation. The band for FFA-OOH was significantly decreased by the incubation as compared with that for PtdCho-OOH. A similar result was obtained from the reaction of a liposomal suspension containing CE-OOH, PtdCho-OOH and LNA-OOH with HDL, in which only LNA-OOH were decreased unequivocally (Fig. 3b). These results suggested that HDL selectively eliminates FFA-OOH in oxidized LDL. The reaction of FFA-OOH with HDL was investigated further by reversed-phase HPLC analyses of the incubation mixture of 13-HPODE or LNA-OOH and HDL. Reversed-phase HPLC analyses demonstrated the appearance of a peak due to 13-HODE by the incubation of 13-HPODE with HDL (Fig. 4a), suggesting that HDL could convert 13-HPODE to 13-HODE through two-electron reduction. It was confirmed by the incubation of LNA-OOH with HDL (Fig. 4b) in which 13-HODE also appeared in the chromatogram after the incubation. LNA-OOH are known to involve equal amounts of 13-HPODE and 9-HPODE conjugated diene isomers [36]. Therefore, the peaks not corresponding to 13-HPODE and 13-HODE in the chromatogram for LNA-OOH were definitively 9-HPODE and 9-HODE, respectively. It is therefore likely that two LNA-OOH isomers were converted to their respective hydroxyoctadecadienoic acid isomers through two-electron reduction by HDL.

### Contribution of ApoA-1 to the NEFA-OOH-Reducing Activity of HDL

**Fig. 5:**
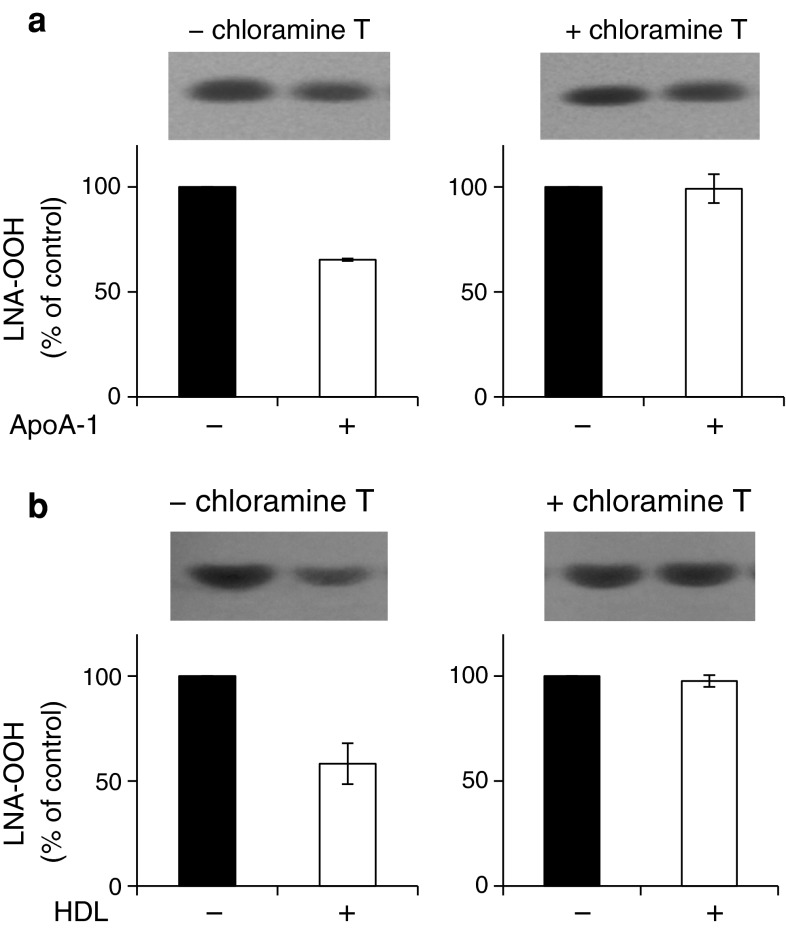
Effect of chloramine-T on the LNA-OOH reducing activity of HDL. **a** Incubation of LNA-OOH with apoA-1, **b** incubation of LNA-OOH with HDL. The liposomal suspension containing LNA-OOH was prepared using the same procedure described above (LNA-OOH; 0.5 mM, DM-PtdCho; 10 mM, cholesterol; 5 mM). To 100 μL of the liposomal solution, 600 μL of apoA-1 solution or HDL solution was added to adjust the final concentration to 0.5 mg protein/mL. For treatment with chloramine-T, the buffer solution of apoA-1 or HDL (1.0 ml/mL) was mixed with chloramine-T (0.5 mM) and incubated at 37 °C for 1 h. Then the chloramine-T-treated solution was added to the 100-μL liposomal suspension to adjust the final volume to 600 μL (final concentration of apoA-1 and HDL: 0.5 mg/mL). After incubation at 37 °C for 6 h, total lipids were extracted and the extract subjected to quantitative normal-phase TLC analyses as described above.

**Fig. 6:**
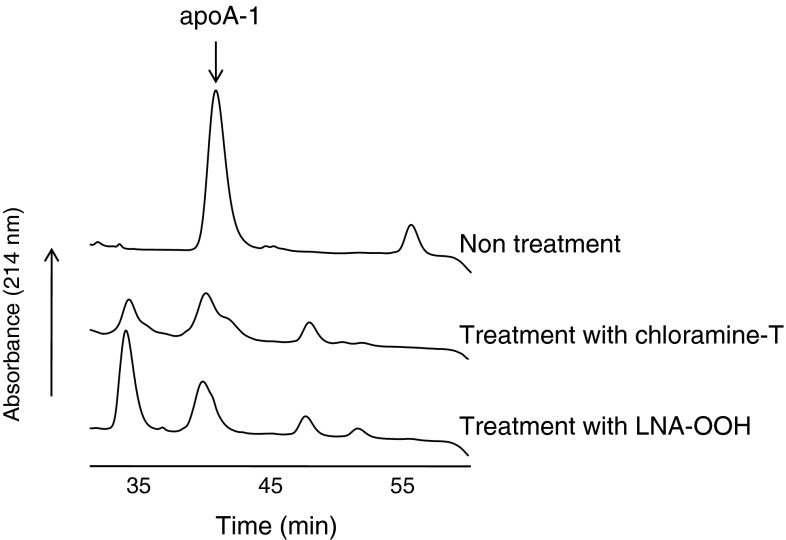
HPLC analyses of HDL after treatment with LNA-OOH or chloramine-T. Treatment with LNA-OOH; HDL (3 mg protein/mL) was incubated with a liposomal suspension containing 25 mM DM-PtdCho and 12.5 mM cholesterol with 1.25 mM LNA-OOH at 37 °C for 24 h. Treatment with chloramine-T; HDL (0.3 mg protein/mL) was treated with chloramine T (30 μM) at 37 °C for 1 h. After the reaction was complete, each sample was applied to a reversed-phase HPLC column of TSK gel ODS-80Ts with a gradient solvent system of solvent A (water containing 0.1 % TFA) and solvent B (acetonitrile containing 0.1 % TFA) in B; 25 % (0–10 min), 25–45 % (10–15 min), 45–55 % (15–47 min), 55–95 % (47–57 min) and 95–100 % (57–58 min) (18). The eluent was monitored by UV absorption at 214 nm at a flow rate of 0.5 mL/min

ApoA-1 has long been suggested to participate in the reduction of LOOH by HDL. This is because apoA-1 is the major apoprotein of HDL and contains methionine residues which are thought to convert LOOH to their respective hydroxyl compounds by the two-electron reduction reaction [37]. Chloramine-T is known to be a methionine-specific oxidant in lipoproteins [38]. Thus, the FFA-OOH-reducing activities of apoA-1 and HDL were estimated with and without treatment with chloramine-T (Fig. 5a and b). The results clearly demonstrated that apoA-1 could reduce LNA-OOH without chloramine-T treatment. Treatment with chloramine-T diminished the LNA-OOH-reducing effect of apoA-1. In addition, the LNA-OOH-reducing effect of HDL was also diminished by treatment with chloramine-T, suggesting that methionine residues in apoA-1 are responsible for the HDL-dependent reduction of FFA-OOH. Next, reversed-phase HPLC analyses were applied for evaluation of the oxidative modification of HDL proteins (Fig. 6). By incubation with LNA-OOH, a main peak at 41 min was decreased and a new peak appeared at 34 min. This change was also reproduced when HDL was treated with chloramine-T. From the comparison with the result of Wang et al. [35], each peak was assigned to intact apoA-1 and oxidized apoA-1, respectively. Therefore, LNA-OOH was presumed to react with the methionine residues of HDL in a similar way to that seen with chloramine-T.

## Discussion

Unsaturated lipids are susceptible to free radical-induced chain oxidation, thereby resulting in the production of LOOH as primary oxidation products. Noguchi et al. [39] found that PtdCho-OOH and CE-OOH were the major LOOH species formed in the free radical-induced chain oxidation of LDL and, relative to PtdCho-OOH, CE-OOH was larger within the LDL. The present study demonstrated that copper ion-induced oxidation of LDL led to the accumulation of CE-OOH in preference to PtdCho-OOH (Fig. 1). This metal-catalyzed oxidation proceeds via a radical chain reaction and is frequently used as an in-vitro model of oxidized LDL formation [40]. Hence, the reason for the preference of CE-OOH in oxidized LDL should be elucidated to understand the molecular mechanism of the pathological effect of oxidized LDL in the arteries. One hypothesis is that PtdCho is more resistant to copper ion-induced radical chain oxidation than CE. More plausible hypothesis is that the reaction products, PtdCho-OOH, are more susceptible to copper ion-catalyzed one-electron reduction to produce short chain carbonyls such as 4-hydroxynonenal, and PAF-like PtdCho core aldehydes with pro-inflammatory activities, resulting in promotion of the atherogenic effect of LDL. In fact, PAF-like PtdCho core aldehydes are known to mediate the signaling pathways of DNA synthesis and production of nitric oxide independently of the activation of the PAF-receptor in vascular smooth muscle cells [7]. LDL and HDL possess phospholipase A_2_ activity that presumably originates from PAF-AH [41]. We found that the PAF-AH activity of LDL was much higher than that of HDL, suggesting that oxidized LDL is liable to release oxidatively modified FFA and lysoPtdCho from PtdCho core aldehydes due to its own PAF-AH activity. Therefore, the phospholipase A_2_ activity of LDL may have a dual rule in atherosclerosis, i.e., clearance of pro-inflammatory PAF-like PtdCho core aldehydes and release of lysoPtdCho, which promotes the recruitment of macrophages into the intima and impairs endothelial cells [41].

The present study demonstrated that the FFA-OOH level was increased in accordance with the formation of lysoPtdCho in copper ion-induced LDL oxidation and that the increase of both compounds was suppressed by the addition of a PAF-AH inhibitor in the reaction mixture (Fig. 2). PAF-AH was reported to involve phospholipase A_2_ action toward phospholipid hydroperoxides [16, 17]. Therefore, the present study confirmed that PtdCho-OOH formed by the radical chain oxidation of PtdCho act as substrates for the phospholipase A_2_ activity of PAF-AH present in LDL. FFA-OOH and lysoPtdCho seem to be released by PAF-AH-dependent hydrolysis of PtdCho-OOH for elimination of this primary oxidation product of PtdCho from oxidized LDL. CE-OOH is unlikely to yield FFA-OOH because CE-OOH is not the substrate for PAF-AH.

Two-electron reduction of LOOH to their hydroxyl derivatives is an alternative elimination mechanism of LOOH accumulation in LDL. Watson et al. [14] suggested that PON-1 attached to HDL reduces peroxidized phospholipids directly to stable hydroxyl derivatives. However, several studies have demonstrated that the hydroperoxy group in LOOH can react with the methionine residue of apoproteins in plasma lipoproteins involving LDL and HDL, thereby resulting in hydroxyl derivatives and methionine sulfoxide by two-electron transfer reactions [18, 36, 42]. This non-enzymatic reduction of LOOH is assumed to be LOOH detoxification and may be involved in the antioxidant ability of HDL in which PtdCho-OOH are assumed to be first transferred to HDL for the reduction of the hydroperoxy group by methionine residues of apoA-1 with the formation of phospholipid hydroxides and methionine sulfoxide [43]. We revisited the two-electron reduction of LOOH by the methionine residue of apoA-1 using oxidized LDL solution and a liposomal suspension containing LNA-OOH, PtdCho-OOH and CE-OOH (Fig. 3): HDL reduced FFA-OOH selectively. Furthermore, this reduction occurred through a two-electron reduction of the hydroperoxy group to the hydroxy group because 13-HPODE was converted to 13-HODE by incubation with HDL. In addition, chloramine-T treatment of HDL and apoA-1 suppressed the reduction of LNA-OOH, suggesting that the methionine residue is responsible for the reduction of FFA-OOH. Also, the HPLC pattern of the reaction mixture of HDL and LNA-OOH corresponded to that obtained from chloramine-T-treated HDL (Fig. 6). It was therefore confirmed that the methionine residue of apoA-1 reacts with FFA-OOH preferably to produce their hydroxyl derivatives. FFA-OOH may be removed from oxidized LDL particles more readily than esterified LOOH because of their lower hydrophobicity. This may be the reason why the methionine residues of apoA-1 react with FFA-OOH selectively.

Taken together, the present study provided a purposive scenario for the antioxidant ability of HDL on oxidized LDL as follows: (i) PtdCho-OOH formed by the radical chain oxidation of the unsaturated acyl group of PtdCho in LDL is gradually hydrolyzed by the phospholipase A_2_ activity of PAF-AH present in LDL; (ii) the resulting FFA-OOH is immediately transferred onto the methionine residue of apoA-1 constituting HDL; (iii) FFA-OOH is converted to stable hydroxyl fatty acids by the reducing activity of the methionine residue. Recent studies suggest that methionine sulfoxide (an oxidation product of methionine from FFA-OOH reduction) is returned to methionine by the action of the methionine sulfoxide reductase system [44, 45]. The “recycling” from methionine sulfoxide to methionine may have a key role in the antioxidant ability of HDL from the viewpoint of the two-electron reduction of LOOH by the methionine residue of apoA-1. Further study on the antioxidant ability of HDL in relation to its anti-atherogenic effect is warranted.

## Abbreviations

LOOH: Lipid hydroperoxides
FFA-OOH: Nonesterified fatty acid hydroperoxides
CE-OOH: Cholesteryl ester hydroperoxides
PtdCho-OOH: Phosphatidylcholine hydroperoxides
LNA-OOH: Linoleic acid hydroperoxides
PAF-AH: Platelet activating factor-acetylhydrolase
apoA-1: Apolipoprotein A-1
PL-PtdCho: 1-Palmitoyl-2-linoleoyl-*sn*-glycero-3-phosphocholine
DM-PtdCho: Dimyristoyl-*sn*-glycero-3-phosphocholine
lysoPtdCho: Lysophosphatidylcholine
13-HPODE: 13-Hydroperoxyoctadecadienoic acid
13-HODE: 13-Hydroxyoctadecadienoic acid
TMPD: *N*,*N*,*N*′,*N*′-tetramethyl-*p*-phenylenediamine dihydrochloride
